# Supplementary material for: Economic burden of chronic pain in Alberta, Canada
Source: PLoS One. 2022 Aug 12;17(8):e0272638. doi: 10.1371/journal.pone.0272638 (PMC9374207; doi:10.1371/journal.pone.0272638)
Supplement: S1 File — (DOCX) [file pone.0272638.s001.docx]

**Supporting information 1**

**S1 Table. Incremental costs of other services per person with chronic pain per year**

|  | **Incremental utilization (#visits)** | | | **Cost per visit*** | **Incremental cost ($)** | | |
| --- | --- | --- | --- | --- | --- | --- | --- |
| **Service** | **Male** | **Female** | **Both** |  | **Male** | **Female** | **Both** |
| Nurse | 0.48 | 0.41 | 0.45 | $ 77.74 | $ 37.32 | $ 31.87 | $ 34.98 |
| Chiropractor | -0.02 | 0.57 | 0.35 | $ 115.00 | $ (2.30) | $ 65.55 | $ 40.25 |
| Physiotherapist | 0.93 | 1.17 | 1.04 | $ 80.00 | $ 74.40 | $ 93.60 | $ 83.20 |
| Psychologist | 0.07 | -0.02 | 0.02 | $ 200.00 | $ 14.80 | $ (4.00) | $ 4.00 |
| Social worker | 0.34 | 0.04 | 0.16 | $ 140.00 | $ 47.60 | $ 5.60 | $ 22.40 |
| Occu.therapist | 0.06 | 0.10 | 0.08 | $ 132.00 | $ 7.92 | $ 12.67 | $ 10.43 |
| **Total** |  |  |  |  | **$ 179.74** | **$ 205.30** | **$ 195.26** |
| *data sources: | Accessed on March 5, 2021 | | | | | | |
| Nurse | <https://www.dr-bill.ca/blog/ahcip/alberta-fee-code-changes-all-physicians> | | | | | | |
| Chiropractor | <https://ourinsurancecanada.com/does-alberta-health-cover-chiropractors/#:~:text=HOW%20MUCH%20IS%20A%20CHIROPRACTOR,the%20Chiropractor%20more%20than%20once.> | | | | | | |
| Physiotherapist | <https://csaphysio.ca/fees> | | | | | | |
| Psychologist | <https://psychologistsassociation.ab.ca/about-paa/resources/recommended-fee-schedule> | | | | | | |
| Social worker | <https://www.asafeplacetogrow.ca/fees-billing-policies> | | | | | | |
| Occupational therapist | <https://www.ualberta.ca/stuttering-speech-therapy/media-library/documents/2017-july-istarinformationpackage.pdf> | | | | | | |

**S2 Table. International Classification of Disease (ICD) codes and methods to estimate costs of the 11 chronic pain (CP)-related conditions.**

| **Conditions** | **ICD-10 codes** | **ICD-9 codes** |
| --- | --- | --- |
| Injury | S, T, V and Y | 800 to 999, and E |
| Cancer | C and D00 to D48 | 140 to 239 |
| Musculoskeletal | M | 710 to 739 |
| Mental disorder | F | 290 to 319 |
| Digestive | K | 520 to 579 |
| Neurological | G | 320 to 389 |
| Infectious | A and B | 1 to 139 |
| Cardiovascular | I | 390 to 459 |
| Genitourinary | N | 580 to 629 |
| Endocrine/hormonal | E | 240 to 279 |
| Respiratory | J | 460 to 519 |

The 11 CP-related conditions were identified by the most responsible diagnosis using chapters of the ICD-10 codes in the Discharge Abstract Database (DAD) and National Ambulatory Care Reporting System (NACRS) database as shown in S2 Table. We estimated health services utilization costs per patient per year by condition for the year of 2019. Conservatively, only those with at least one hospitalization or one outpatient visit were considered patients. Using the case mix group plus (CMG+) methods, we estimated costs for their utilizations of inpatient and outpatient services recorded in DAD and NACRS [14,15]. Linking their unique lifetime identifiers (ULIs) to the pharmaceutical information network (PIN), practitioner claims and diagnostic imaging (DI) databases, we additionally extracted their utilizations of prescription drugs, practitioner and DI services, respectively, for the same year 2019. We used the ICD-9 codes (S2 Table) to identify practitioner visits and estimate costs (the paid amount in claims) associated with the 11 CP-related conditions. For prescription drugs and DI services, we included all dispensations and DI exams in 2019 that were associated with each patient’s ULI. Costs for prescription drugs were based on the Alberta Drug Benefit List [16] and costs for DI were retrieved from Department of Finance, Alberta Health Services.

**S3 Table. HSU costs of CP-related conditions, weights, and incremental healthcare cost per person with CP in Alberta**

| **Condition** | **Cost** | **Weight*** | **Weighted cost** |
| --- | --- | --- | --- |
| Injury | $ 3,391.33 | 0.552 | $ 1,873.12 |
| Cancer | $ 9,401.19 | 0.023 | $ 218.63 |
| Musculoskeletal | $ 5,237.31 | 0.350 | $ 1,834.58 |
| Mental health/behavioural | $ 13,057.32 | 0.016 | $ 208.77 |
| gastrointestinal | $ 6,196.45 | 0.015 | $ 90.06 |
| Neurological | $ 8,771.89 | 0.010 | $ 89.25 |
| Infection | $ 4,258.65 | 0.009 | $ 37.14 |
| Circulatory (CVD) | $ 3,990.68 | 0.010 | $ 40.60 |
| Genitourinary | $ 4,466.09 | 0.009 | $ 38.95 |
| Endocrine/hormonal | $ 7,052.19 | 0.003 | $ 20.50 |
| Respiratory | $ 4,268.42 | 0.003 | $ 12.41 |
| **HSU cost per person with chronic pain (I)** |  |  | **$ 4,464.01** |
| **HSU cost per average person in the population (II)** | | | $ 2,442.00 |
| **Incremental HSU cost per person with chronic pain (III=I-II)** | |  | **$ 2,022.01** |
| **Incremental other service cost per person with chronic pain (IV)** | |  | $ 195.26 |
| **Total incremental healthcare cost (V=III+IV)** |  |  | **$ 2,217.27** |
| (I) includes costs for inpatient, outpatient, physician, DI, and prescription drugs estimated from the AH admin. databases | | | |
| (II) was obtained from Thanh et al 2014 and inflated to 2020 [17]. | | | |
| (IV) includes costs for nurse, physiotherapist, chiropractor, psychologist, social worker, and occupational therapist services estimated from the CCHS data | | | |
| *Weight was from Painaustralia report [6] after the unknown was proportionately distributed to the other conditions | | | |

**S4 Table. Model inputs to estimate costs of lost productivity**

| **Variable** | **Chronic pain, mean (95%CI)** | | **P-value** |
| --- | --- | --- | --- |
|  | **Yes** | **No** |  |
| Percentage of unable to work | 10.21%  (7.62% to 12.8%) | 1.74%  (1.17% to 2.31%) | <0.001 |
| Percentage of unemployment | 9.89%  (7.2% to 12.59%) | 9.56%  (8.26% to 10.85%) | 0.411 |
| Number of missed working days per 3 months | 1.74  (1.06 to 2.41) | 0.28  (0.16 to 0.40) | <0.001 |
| Number of missed working hours per week* | 4.47  (3.13 to 5.81) | 1.7  (1.19 to 2.21) | <0.001 |
| All variables were estimated from the CCHS data except * which was a weighted average estimated from the data of Kawai et al. [19] with a 95%CI based on an assumed variation of ±30%. | | | |
|  | |  |  |

**S5 Table. Number of people (population) by age and sex in Alberta [8]**

| **Age group** | **Male** | **Female** | **Total** |
| --- | --- | --- | --- |
| 12 to 17 years* | 160,378 | 153,325 | 313,702 |
| 18 to 64 years* | 1,428,502 | 1,383,178 | 2,811,680 |
| 65 years or older | 270,342 | 310,050 | 580,392 |
| All ages | 1,859,221 | 1,846,553 | 3,705,774 |

*Assumed the number is distributed equally for each age between 10 and 14 and between 15 and 19.

**S6 Table. Incremental cost of productivity losses per person with chronic pain**

| **Types of analysis** | **Chronic pain** | | **Incremental** |
| --- | --- | --- | --- |
|  | **Yes** | **No** |  |
| Base-case analysis (Average) | $ 16,960 | $ 8,548 | $ 8,412 |
| Deterministic sensitivity analysis |  |  |  |
| Low | $ 11,872 | $ 5,984 | $ 5,888 |
| High | $ 22,048 | $ 11,113 | $ 10,935 |
| Probabilistic sensitivity analysis |  |  |  |
| Low | $ 9,671 | $ 4,344 | $ 5,327 |
| High | $ 24,262 | $ 12,765 | $ 11,498 |
