# Supplementary figures and images for: Economic burden of chronic pain in Alberta, Canada
Source: PLoS One. 2022 Aug 12;17(8):e0272638. doi: 10.1371/journal.pone.0272638 (PMC9374207; doi:10.1371/journal.pone.0272638)

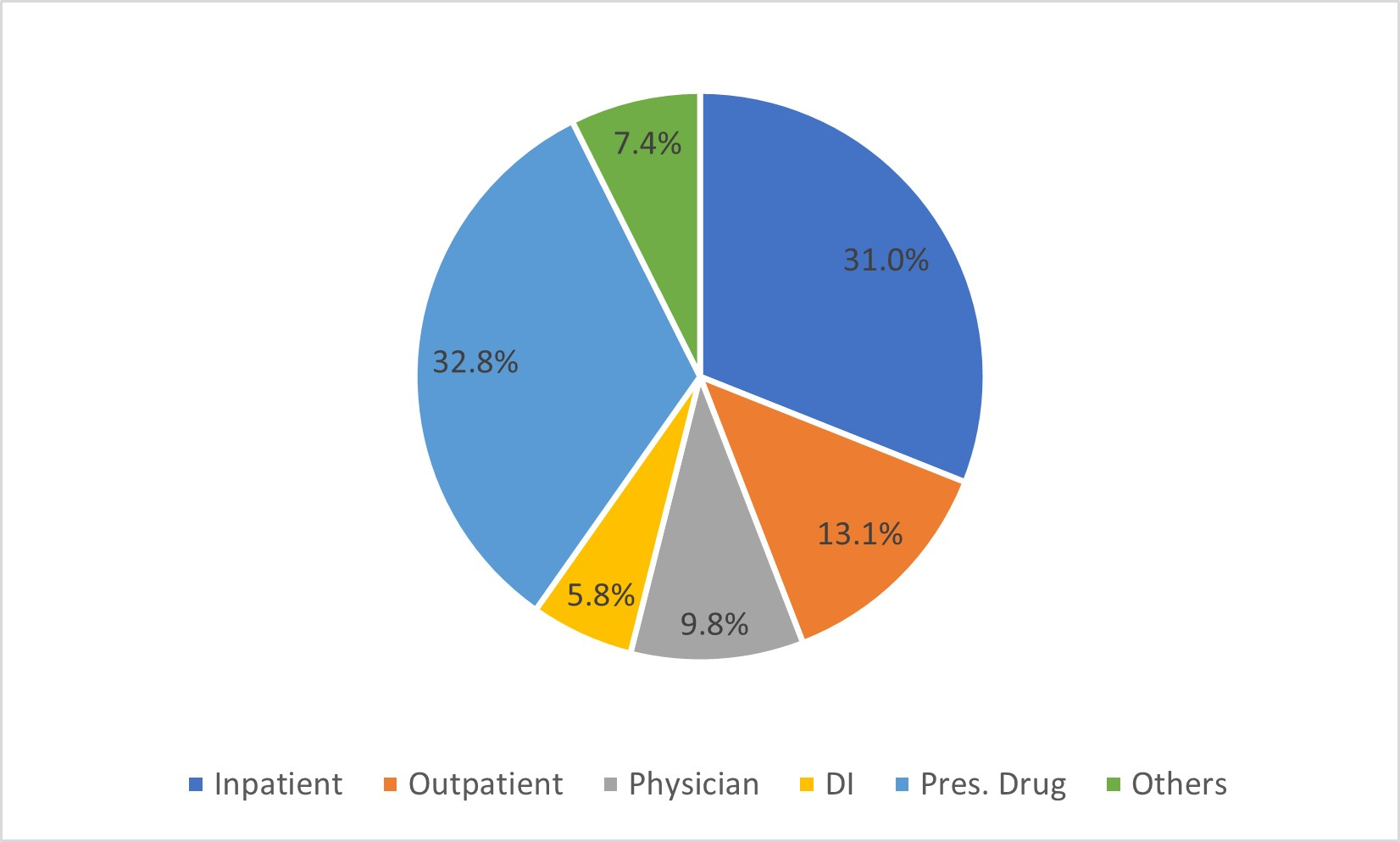

Supplement: S1 Fig — (TIF) [file pone.0272638.s002.tif]
