# Supplementary material for: Economic burden of chronic pain in Alberta, Canada
Source: PLoS One. 2022 Aug 12;17(8):e0272638. doi: 10.1371/journal.pone.0272638 (PMC9374207; doi:10.1371/journal.pone.0272638)
Supplement: Attachment — Submitted filename: PONE-D-22-11788_reviewer comments.docx [file pone.0272638.s003.docx]

**Economic Burden of Chronic Pain in Alberta, Canada**

The manuscript “Economic Burden of Chronic Pain in Alberta, Canada” by Thanh et al. estimated the incremental costs of chronic pain (CP) in Alberta, Canada from a societal perspective. The incremental costs, hence, included healthcare costs and productivity losses. The number of persons with CP was estimated from Canadian Community Health Survey (CCHS) and the incremental healthcare costs per person with CP (compared to non-CP) were estimated from Alberta administrative health data. The productivity loss was estimated from modelling, using data from the CCHS and published data from literature.

Overall, the manuscript is well structured, nicely written, and easy to follow. The methods for estimating the costs (both healthcare and productivity loss) are robust. The study implication is important as its findings will serve the Alberta Pain Strategy 2019-2024.

I have a couple of comments and questions as below:

1. By definition, CP is long standing pain that persists beyond usual recovery period (3 months) as in the introduction. However, the questions of pain and discomfort in the CCHS did not specify time period of pain/discomfort. Therefore, it could be the case that the prevalence using CCHS would be overestimated. Although, in the discussion, the authors cited another study which reported similar prevalence (20.6%), the authors should discuss potential bias of using the CCHS prevalence where a strict definition of CP was not used.
2. Several components of healthcare costs (inpatient, outpatient, physician claims, diagnostic imaging, and drug costs) were estimated from administrative health data though 11 proxy CP-related conditions. However, it’s not clear how these conditions were included. The authors should provide more details on
   a. ICD codes for these conditions, probably in Supplementary documents
   b. How these conditions (primary diagnosis vs. secondary diagnoses) in inpatient, outpatient, and physicians claims database were included for cost calculation?
   c. How the authors identified what diagnostic imaging was for CP patients? Is there a timeframe between an inpatient stay/ outpatient visit/ physician claim with a diagnosis of CP-related conditions and an imaging event to determine that the imaging event was actually for the CP-related conditions?
   d. What drugs were included for CP-related drug costs?
