# Supplementary material for: Economic burden of chronic pain in Alberta, Canada
Source: PLoS One. 2022 Aug 12;17(8):e0272638. doi: 10.1371/journal.pone.0272638 (PMC9374207; doi:10.1371/journal.pone.0272638)
Supplement: Attachment — Submitted filename: Response to Reviewers.docx [file pone.0272638.s004.docx]

**Response to Reviewers**

Reviewer #1: Economic Burden of Chronic Pain in Alberta, Canada
The manuscript “Economic Burden of Chronic Pain in Alberta, Canada” by Thanh et al. estimated the incremental costs of chronic pain (CP) in Alberta, Canada from a societal perspective. The incremental costs, hence, included healthcare costs and productivity losses. The number of persons with CP was estimated from Canadian Community Health Survey (CCHS) and the incremental healthcare costs per person with CP (compared to non-CP) were estimated from Alberta administrative health data. The productivity loss was estimated from modelling, using data from the CCHS and published data from literature.
Overall, the manuscript is well structured, nicely written, and easy to follow. The methods for estimating the costs (both healthcare and productivity loss) are robust. The study implication is important as its findings will serve the Alberta Pain Strategy 2019-2024.
I have a couple of comments and questions as below:
1. By definition, CP is long standing pain that persists beyond usual recovery period (3 months) as in the introduction. However, the questions of pain and discomfort in the CCHS did not specify time period of pain/discomfort. Therefore, it could be the case that the prevalence using CCHS would be overestimated. Although, in the discussion, the authors cited another study which reported similar prevalence (20.6%), the authors should discuss potential bias of using the CCHS prevalence where a strict definition of CP was not used.

As suggested, we have added this as a limitation on page 17.

2. Several components of healthcare costs (inpatient, outpatient, physician claims, diagnostic imaging, and drug costs) were estimated from administrative health data though 11 proxy CP-related conditions. However, it’s not clear how these conditions were included. The authors should provide more details on
a. ICD codes for these conditions, probably in Supplementary documents

b. How these conditions (primary diagnosis vs. secondary diagnoses) in inpatient, outpatient, and physicians claims database were included for cost calculation?
c. How the authors identified what diagnostic imaging was for CP patients? Is there a timeframe between an inpatient stay/ outpatient visit/ physician claim with a diagnosis of CP-related conditions and an imaging event to determine that the imaging event was actually for the CP-related conditions?
d. What drugs were included for CP-related drug costs?

As suggested, we have included a Supplement Table S2 containing all the above-mentioned information.

Reviewer #2: I would like to thank authors for writing an interesting article. In general, this is a well written and analyzed study. Chronic pain (CP) is highly prevalent, so it is important study topic. However, it is a difficult one, since there is very few accurate methods to estimate costs related to CP. In this study, the authors used methods that were mainly developed and used before, so from the methodological point of view there was not much new in this study. If there are, I would suggest authors to address them more clearly.

We agree with reviewer that the methods have been used previously. As ICD-10 codes of CP were not used, costs of CP were not directly estimated, but indirectly through its’ related conditions. We have mentioned this as a limitation and suggested further study when ICD-11 codes of CP has been used widely (page 17)

Some comments and suggestions:
1) Abstract; could you add to the methods the time interval that you used in your results section. You only once say it is one year. Timeframe is important once we interpret the results.

We have revised as suggested

2) Introduction; It would be good to add one or two sentences about Alberta and its population and healthcare, since Alberta is not very well known internationally.

As suggested, we have added “(a western province of Canada with a population of approximately 4.4 million and a publicly funded healthcare system)” to page 5.

3) Also you may say shortly what kind of objectives / interventions the provincial pain strategy is planning to implement. At the current text, there is not much link between the economic outcome and the potential interventions. Are they aiming more for prevalence of CP by reducing the severity/intensity of the pain, or is it trying to reduce chronic diseases where CP is associated (as used in the study methods).

We have already referred to reference #9 for readers who want to know more about Alberta Pain Strategy. However, as suggested by reviewer, to make it clearer, we have now added “aiming to support people living with CP, their families/caregivers, and providers to optimize management of CP and its’ impacts on individuals’ function and quality of life, as well as on societal economic burden” to page 6.

4) The study uses CCHS survey data that is pretty comprehensive and representative for the Alberta population. However, it is self reported. At least for some key diseases there are Canadian estimates for prevalence and incidence. I would have liked to see more comparison of your estimates to existing literature. Related to this, you should discuss more in detail, how accurate the survey question for the used 12 CD from Australian study are, since the question asks "have your physician even told you have X disease". So it is not really close estimate from prevalence or incidence.

In our study, CCHS was used for CP prevalence only. As it is subjective and not specific to pain persisting for more than 3 months (as the definition), we have already discussed this as a limitation (page 17). The 11 CP-related conditions were identified by ICD-10 codes in the Alberta Health Administrative Databases. We have now added a supplement Table S2 to clarify this further as also suggested by reviewer 1.

5) In the results you have several small tables that are (almost) fully reported also in the text. You could easily reduce the number of tables in the main text either just showing some results in text only or combining some of them. Maybe you then could bring one table from suppl material to main text.

As suggested, we have combined Tables 3 and 4 together, so that there is a total of 4 tables and 1 figure in the main text now. We did not bring any table from supplement to keep the main text simpler and easier to follow.

6) You mention that the labor variable was from Newfoundland Labrador (NL). I found that rather strange since the NL and Alberta are pretty different considering the labor force (age, unemployment, education, occupation). I suggest that you discuss the validity of this data use in the discussion section.

We have already discussed this in the final limitation (page 17).

7) You used the Australian method to estimate CP in CDs. I was not sure if this method is really accurate enough. Also related to point 3 above, this method only finds change if the prevalence is changing, unless you can change the ratios. Could you discuss this considering the measurement after the pain program cost estimation.

We have discussed this in the first limitation and suggested further study when ICD-11 codes of CP have been used widely (page 17).

------------------------------
